# Anti-apoptotic role of HIF-1 and AP-1 in paclitaxel exposed breast cancer cells under hypoxia

**DOI:** 10.1186/1476-4598-9-191

**Published:** 2010-07-13

**Authors:** Lionel Flamant, Annick Notte, Noelle Ninane, Martine Raes, Carine Michiels

**Affiliations:** 1 Laboratory of Biochemistry and cellular Biology (URBC), NARILIS - NAmur Research Institute for LIfe Sciences - FUNDP-University of Namur, 61 rue de Bruxelles, 5000 Namur, Belgium

## Abstract

**Background:**

Hypoxia is a hallmark of solid tumors and is associated with metastases, therapeutic resistance and poor patient survival.

**Results:**

In this study, we showed that hypoxia protected MDA-MB-231 breast cancer cells against paclitaxel- but not epirubicin-induced apoptosis. The possible implication of HIF-1 and AP-1 in the hypoxia-induced anti-apoptotic pathway was investigated by the use of specific siRNA. Specific inhibition of the expression of these two transcription factors was shown to increase apoptosis induced by chemotherapeutic agents under hypoxia indicating an involvement of HIF-1 and AP-1 in the anti-apoptotic effect of hypoxia. After HIF-1 specific inhibition and using TaqMan Human Apoptosis Array, 8 potential HIF-1 target genes were identified which could take part in this protection. Furthermore, Mcl-1 was shown to be a potential AP-1 target gene which could also participate to the hypoxia-induced chemoresistance.

**Conclusions:**

Altogether, these data highlight two mechanisms by which hypoxia could mediate its protective role via the activation of two transcription factors and, consecutively, changes in gene expression encoding different anti- and pro-apoptotic proteins.

## Background

Hypoxia, a reduction in the normal level of tissue oxygen tension, is well evidenced within most of solid tumors [1]. Forty percent of all breast cancers are reported to present hypoxic region (for a review [2]) and tumor hypoxia is often associated with resistance to chemotherapy, immunotherapy and radiotherapy [3-5]. It is now apparent that hypoxia, according to its severity, can either promote apoptosis and cell death or contrariwise prevent cell death by provoking an adaptive response leading to cell proliferation and tumor growth. The hypoxic environment which takes place in the centre of solid tumors leads to a variety of biological responses [6,7], the activation of the transcription factor HIF-1 (Hypoxia Inducible Factor-1) being a major one [8]. HIF-1 is an heterodimer composed of two subunits belonging to the bHLH-PAS family [9], i.e. the hypoxic response factor HIF-1α and the nuclear constitutively expressed HIF-1β subunit, also called ARNT (aryl hydrocarbon receptor nuclear translocator). In the presence of oxygen, HIF-1α is hydroxylated on two prolines (residues 564 and 402) by specific oxygen prolyl hydroxylases (PHD). This hydroxylated form can then interact with the von Hippel-Lindau protein (pVHL), which is part of an E3 ubiquitin ligase complex, thus targeting this subunit for degradation by the proteasome [10,11]. A second regulatory mechanism involves the hydroxylation of the asparagine 803 by an oxygen-dependent asparaginyl hydroxylase (FIH-1) which prevents the interaction between HIF-1α and its coactivators p300 and CBP [12]. Under hypoxic conditions, HIF-1α is no longer modified, which results in its stabilization and its translocation into the nucleus. It then dimerizes with HIF-1β, forming the active HIF-1 transcription factor capable of binding to the specific sequence called HRE (hypoxia response element) present in the promoter of its target genes [13].

Many studies demonstrate that HIF-1, activated by hypoxia, could play an anti-apoptotic role by regulating the expression of anti- and pro-apoptotic proteins [14-16]. However, hypoxia was also described to play an anti-apoptotic role by regulating expression of anti- and pro-apoptotic proteins independently of HIF-1 [17]. Besides HIF-1, number of other transcription factors could be activated by hypoxic environment, among which AP-1, already described to be implicated in the hypoxia-induced protection against chemotherapeutic-induced apoptosis [18].

If the mechanisms underlying the hypoxia-induced radio- and chemoresistance begin to be described, the actual actors of the protection still need to be identified. The aim of this study was (i) to investigate the effect of hypoxia on apoptosis induced by two chemotherapeutic agents in human breast cancer MDA-MB-231 cell line and (ii) to characterize the mechanisms underlying the hypoxia resistance against paclitaxel-induced apoptosis observed in these cells by investigating the putative implication of the two transcription factors HIF-1 and AP1.

We used the two chemotherapeutics agents paclitaxel and epirubicin as apoptosis inducers. These two drugs are some of the most active agents in the treatment of breast cancer [19]. Paclitaxel, belonging to the taxane group of chemotherapeutic agents, binds to microtubules and causes their stabilization, inducing cell cycle arrest at G2/M mitotic phase followed by apoptosis. It also induces modulation expression or posttranslational modification of some pro- and anti-apoptotic proteins and regulation modification of several enzymes involved in the regulation of apoptosis [20,21]. Epirubicin HCl, an anthracycline analog, derivative of doxorubicin, intercalates into DNA strands, inhibiting DNA and RNA synthesis. It inhibits the progression of topoisomerase II by stabilizing DNA-topoisomerase complex, resulting in DNA damage and induction of apoptosis [22]. It also induces formation of reactive oxygen species promoting apoptosis [23]. The biochemical and molecular mechanisms of apoptosis activation for these two drugs are complex, affect a number of cellular pathways, and are still under investigation.

## Results

### Hypoxia protects MDA-MB-231 cells against paclitaxel-induced apoptosis

**Figure 1:**
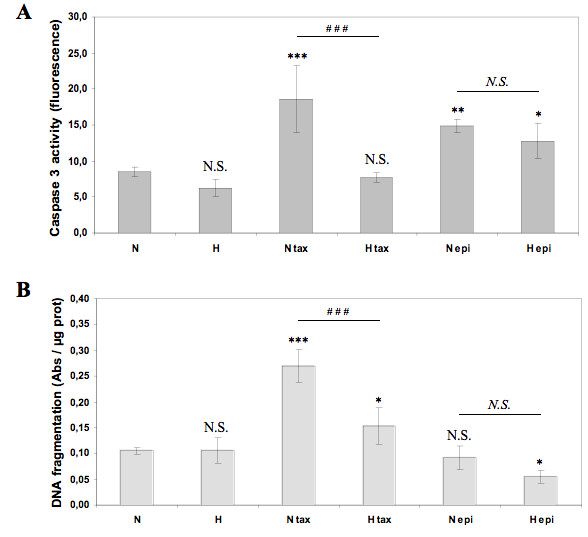
**Effect of hypoxia on the paclitaxel or epirubicin-induced apoptosis**. MDA-MB-231 cells were incubated under normoxic (N) or hypoxic (H) conditions with or without paclitaxel (tax, 50 μM) or epirubicin (epi, 10 μM) for 16 hours. (A) The caspase 3 activity was assayed by measuring free AFC released from the cleavage of the caspase 3 specific substrate Ac-DEVD-AFC. Results are expressed in fluorescence intensity, as mean ± 1 SD (n = 3). (B) After the incubation, DNA fragmentation was assayed using an ELISA for soluble nucleosomes (Cell Death Detection Elisa, Roche). Results are expressed as mean ± 1 SD (n = 3). N.S. = non significantly different from control, * = significantly different from control (p < 0.05), ** = significantly different from control (p < 0.01), *** = significantly different from control (p < 0.001); N.S. = no significant difference between N epi and H epi, ### = significant difference between N tax and H tax (p < 0.001).

Recently, differential effects of hypoxia on drug-induced apoptosis according to the cancer cell lines have been reported and many studies showed that hypoxia can confer resistance against chemotherapy-induced apoptosis in numerous solid tumors including breast cancer [15,18,24]. In order to investigate the effect of hypoxia on paclitaxel- or epirubicin-induced apoptosis, MDA-MB-231 breast cancer cells were incubated under normoxia or hypoxia with or without these two drugs and caspase 3 activity and DNA fragmentation were assessed (Figure 1). Hypoxia per se did not induce apoptosis since no increase in caspase 3 activity or DNA fragmentation were observed after 16 hours incubation. Paclitaxel at 50 μM and epirubicin at 10 μM did trigger apoptosis as shown by an increase in caspase 3 activity in normoxic conditions. Hypoxia did not modify the epirubicin-induced apoptosis. However, it markedly inhibited the paclitaxel-increase in caspase 3 activity. An increase in DNA fragmentation (Figure 1) as well as of nucleus fragmentation as evidenced by DAPI staining (data not shown), other important features of apoptotic cells, were also observed in the presence of paclitaxel under normoxia, which were significantly decreased by hypoxia. The fact that epirubicin showed no DNA fragmentation while caspase 3 was activated may be due to a different kinetics: caspase 3 is indeed activated before DNA fragmentation occurs so that it may occur later than 16 hours. In addition, in order to investigate whether apoptosis is really involved in taxol-induced cell death, the effect of a pan-caspase inhibitor has been studied on overall cell death. Overall cell death has been assessed by quantifying LDH release. Results showed that zVAD-fmk did inhibit taxol- and epirubicin-induced cell death (data not shown). This inhibition was however partial probably because in cell culture, when apoptosis is inhibited, cells die through necrosis, thus also increasing LDH release. Altogether, these data demonstrate that hypoxia is able to protect MDA-MB-231 cells against the chemotherapeutic agent paclitaxel-induced apoptosis.

### Paclitaxel induces modifications in microtubule network and Bim localization

**Figure 2:**
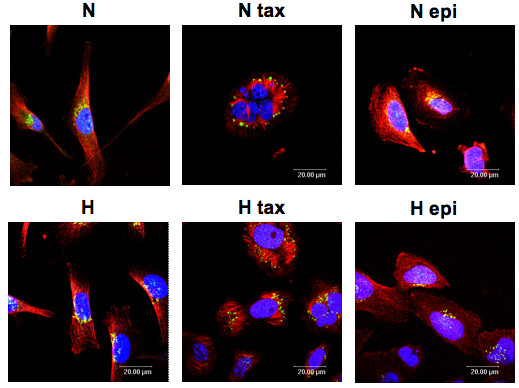
**Effect of hypoxia and/or paclitaxel or epirubicin on Bim protein expression**. MDA-MB-231 cells were incubated under normoxic (N) or hypoxic (H) conditions with or without paclitaxel (tax, 50 μM) or epirubicin (epi, 10 μM) for 16 hours. Bim was detected by immunofluorescence using specific Bim antibody (in green) and α-tubulin antibody was used to stain microtubules (in red). Nuclei were stained with Topro-3 (in blue). Observation was performed in semi-quantitative confocal microscopy.

In order to ensure that the hypoxia protection against paclitaxel was not due to an inactivation of the drug in hypoxic conditions, the paclitaxel effect on microtubules was investigated by immunofluorescence. The localization of the pro-apoptotic protein Bim was also studied (Figure 2). The antimicrotubule drug paclitaxel is known to interfere with microtubule function by binding to the tubulin polymer and stabilizing it against depolymerization. Figure 2 shows that on one hand neither epirubicin, nor hypoxia alone influenced the network of microtubules. On the other hand, in the presence of paclitaxel, we observed a strong modification of the microtubules which became shorter and thicker to form bundles. Hypoxia did not modify this paclitaxel-induced modification of the microtubule network. In the presence of paclitaxel, both under normoxia and hypoxia, we also observed a redistribution of the protein Bim from a perinuclear localization to clusters localized at the microtubule extremities. We thus concluded that hypoxia did not influence the effect of paclitaxel which remains active in hypoxic conditions since microtubule rearrangement and Bim translocation were still observed.

### HIF-1 is involved in the hypoxia-induced protection against paclitaxel-induced apoptosis

Recently, the influence of hypoxia in chemoresistance has been recognized. Hypoxic conditions elicit cellular responses designed to improve cell survival through an adaptive process. The main transcriptional factor activated under hypoxic conditions is HIF-1 (hypoxia-inducible factor-1). Regulation of gene expression through HIF-1 (hypoxia-inducible factor-1) but also via other transcription factors plays an important role in this process. We investigated here whether HIF-1 could be implicated in the hypoxia-induced protection against the apoptosis induced by paclitaxel.

**Figure 3:**
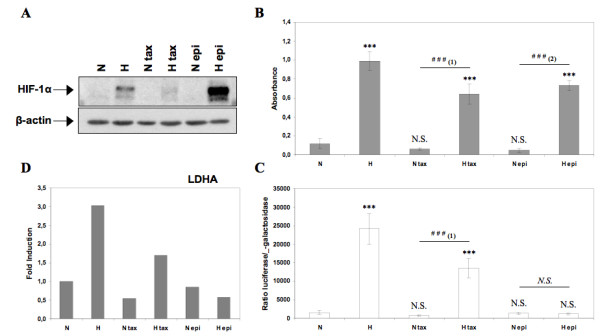
**Analysis of HIF-1α protein expression, HIF-1 DNA binding activity, HIF-1 transcriptional activity and gene expression level of HIF-1 inducible gene LDHA**. MDA-MB-231 cells were incubated under normoxic (N) or hypoxic (H) conditions with or without paclitaxel (tax, 50 μM) or epirubicin (epi, 10 μM) for 16 hours. (A) HIF-1α was detected in total cell extracts by western blotting, using specific antibody. β-actin was used to assess the total amount of proteins loaded on the gel. (B) After the incubation, nuclear extracts were performed and hybridized in the ELISA well containing specific DNA probes (TransAM assay). Detection was performed using an anti-HIF-1α antibody. Results are expressed in absorbance, as mean ± 1 SD (n = 3). (C) Cells were co-transfected with the pUAS-tk-Luc reporter plasmid encoding the firefly luciferase and the pCMVβ normalisation plasmid before incubated. Results are expressed as mean of the ratio between firefly luciferase activity and the β-galactosidase activity ± 1 SD (n = 3). (D) After incubation, total RNA has been extracted and retro-transcribed in cDNA. A real time PCR has been performed with specific primers for LDHA and for RPL13A, a house-keeping gene. Results are expressed in induction level by comparison with the reference condition, normoxia. N.S. = non significantly different from control, *** = significantly different from control (p < 0.001); *N.S. *= no significant difference between N epi and H epi, ### = significant difference between (1) N tax and H tax or (2) N epi and H epi (p < 0.001).

We first evaluated the stabilization of the sub-unit HIF-1α. HIF-1 DNA binding activity as well as transcriptional activity using respectively an ELISA like TransAM assay and a reporter assay and by following the mRNA level of the HIF-1 inducible gene LDHA. Figure 3 shows that hypoxia induced an increase in HIF-1α stabilization, that was lower in the presence of paclitaxel and much higher in the presence of epirubicin compared to the control cells (Figure 3A). DNA binding activity of HIF-1α was increased in hypoxic conditions and neither paclitaxel nor epirubicin influenced this activity (Figure 3B). The transcriptional activity of HIF-1 was not modified by taxol or epirubicin under normoxic conditions while it was increased by hypoxia alone or combined with paclitaxel but not epirubicin. This was directly determined by reporter assay but also indirectly by studying gene expression of a HIF-1 inducible gene, LDHA (Figure 3C, D). HIF-1 thus remains active in presence of paclitaxel under hypoxic conditions. It has to be noted that under hypoxia, epirubicin inhibited HIF-1 activity while HIF-1alpha protein level was upregulated. We hypothesized that the cell "tried" to compensate for not having a HIF-1 active by upregulating further HIF-1alpha level. The mechanism for this has not been studied. The inhibitory effect of epirubicin on HIF-1 was also observed by Semenza's team in human hepatocellular carcinoma cell line Hep3B and in human embryonic kidney cell line HEK293 [25].

In order to study the implication of HIF-1 in the hypoxia anti-apoptotic effect, we then used siRNA directed against the HIF-1α sub-unit. Additional file 1 shows that a concentration of 50 nM of siRNA was high enough to specifically inhibit the synthesis and activity of HIF-1α but not HIF-2α while the RISC-Free control siRNA had no effect.

**Figure 4:**
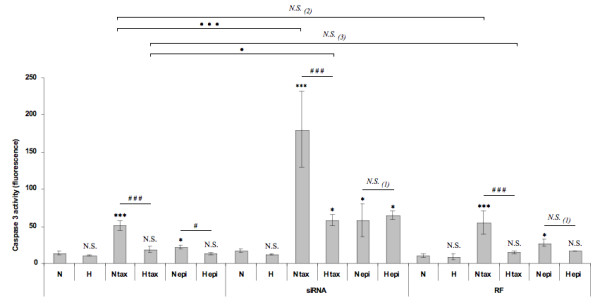
**Effect of HIF-1α silencing on the protective role of hypoxia on the paclitaxel-induced apoptosis**. 8 h post transfection with anti-HIF-1α siRNA (siRNA) or RISC-free control siRNA (RF) (50 nM, 24 h), MDA-MB-231 cells were incubated under normoxic (N) or hypoxic (H) conditions with or without paclitaxel (tax, 50 μM) or epirubicin (epi, 10 μM) for 16 hours. After transfection and incubation, the caspase 3 activity was assayed by measuring free AFC released from the cleavage of the caspase 3 specific substrate Ac-DEVD-AFC. Results are expressed in fluorescence intensity, as mean ± 1 SD (n = 3). Statistical analysis were determined independently for the 3 subgroups *without siRNA*, *with anti-HIF-1α siRNA (siRNA) *and *with RISC-free control siRNA (RF) *; N.S. = non significantly different from control (N, N siRNA or N RF), * = significantly different from control (p < 0.05), *** = significantly different from control (p < 0.001); *N.S. *(1) = no significant difference between N epi and H epi, ### = significant difference between N tax and H tax (p < 0.001), # = significant difference between N epi and H epi (p < 0.05). *N.S. *= no significant difference between (2) N tax and N tax RF or (3) H tax and H tax RF, • = significant difference between H tax and H tax siRNA (p < 0.05), ••• = significant difference between N tax and N tax siRNA (p < 0.001).

Cells were transfected with anti-HIF-1α or control siRNA before being incubated under normoxia or hypoxia in the presence of one or the other agent. The activity of the caspase 3 was then assessed. If HIF-1 plays an anti-apoptotic role, an increase in the paclitaxel-induced caspase 3 activity is expected when inhibited by specific siRNA. Figure 4 shows that, on one hand, the negative control siRNA did not influence caspase 3 activity. On the other hand, this activity was markedly increased for HIF-1α siRNA transfected cells incubated under normoxia as well as under hypoxia, with paclitaxel or epirubicin. No modification was observed in control cells. The caspase 3 activity measured for HIF-1α siRNA transfected cells incubated under hypoxia with paclitaxel reached similar level than the activity measured for non transfected cells incubated in normoxia with paclitaxel, indicating that the protection was reversed in this case.

These results clearly demonstrate an implication of HIF-1 in the hypoxia-induced protection against the apoptosis induced by paclitaxel.

### HIF-1 regulates the expression of pro- and anti-apoptotic genes

Apoptosis is a complex event controlled by numerous pro- and anti-apoptotic genes. In order to understand by which mean HIF-1 protects MDA-MB-231 cells against paclitaxel-induced apoptosis, we studied the expression of 93 genes involved in apoptosis in cells transfected or not with HIF-1α siRNA or with control siRNA and incubated with or without paclitaxel under normoxic or hypoxic conditions, using Taqman Human Apoptosis Arrays.

**Figure 5:**
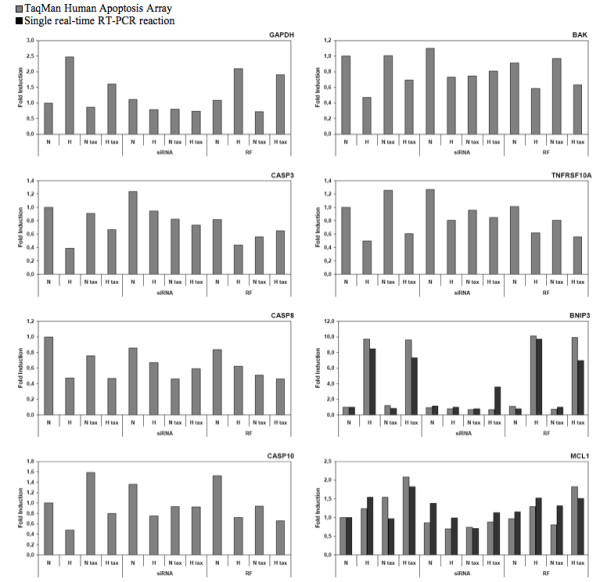
**Gene expression profiling, for genes involved in regulating apoptosis, in HIF-1α silenced MDA-MB-231 cells incubated with or without paclitaxel under normoxic or hypoxic conditions**. 8 h post transfection with HIF-1α siRNA (siRNA) or RISC-free control siRNA (RF) (50 nM, 24 h), cells were incubated under normoxic (N) or hypoxic (H) conditions with or without paclitaxel (tax, 50 μM) for 16 hours. After transfection and incubation, total RNA has been extracted and retro-transcribed in cDNA. TaqMan Human Apoptosis Array (Applied Biosystems) was used to perform real time PCR for 93 human genes implicated in apoptosis in addition to three endogenous controls. 18S was used as the house-keeping gene for data normalization. To validate results, single real-time RT-PCR reaction for BNIP3 and MCL1 were performed for independent samples. Results are expressed in induction level by comparison with the reference condition, normoxia. Please refer to supplementary data (Additional file 1) for results obtained for the 77 genes detected after real time PCR reactions.

Gene expression data for the 77 genes detected after real time PCR reactions are presented in additional file 2. Figure 5 shows gene expression modifications for 8 of these genes which expression seemed to be regulated by HIF-1 and that could be involved in the hypoxia-induced protection against apoptosis induced by paclitaxel.

Some of the genes are well known HIF-1 target genes such as GAPDH and BNIP3 whose expression was enhanced in non transfected cells or cells transfected with control siRNA and incubated under hypoxia with or without paclitaxel and which was inhibited by HIF-1α siRNA. Although BNIP3 is known to play pro-apoptotic function, a putative role in autophagy is more recently conferred to this protein which could thus participate to the hypoxia protection [26,27]. BAK, CASP3, CASP8, CASP10 and TNFRSF10A are five pro-apoptotic genes for which we observed a lower expression under hypoxic conditions when compared to normoxic conditions, for non-transfected cells and cells transfected with the control siRNA. The downregulation of these genes by hypoxia was inhibited when cells were transfected with HIF-1α siRNA, and particularly in the presence of paclitaxel. In this case, hypoxia alone did not influence expression of these genes. We can therefore conclude that HIF-1 is responsible for the decrease of expression of these five pro-apoptotic genes, which could also participate to the hypoxia protection.

Hypoxia also increased the expression of the anti-apoptotic gene MCL-1. siRNA directed against HIF-1α inhibited this effect indicating that HIF-1 could participate to the anti-apoptotic effect of hypoxia by increasing MCL-1 expression under hypoxia in cells incubated in the presence of paclitaxel.

In order to validate our data, we also performed single SYBR Green quantitative real time PCR assays for two of the selected genes (Figure 5). Good correlation between relative transcript abundance data obtained by micro fluidic card and by real time RT-PCR was observed.

### AP-1 is involved in the hypoxia-induced protection against paclitaxel-induced apoptosis

**Figure 6:**
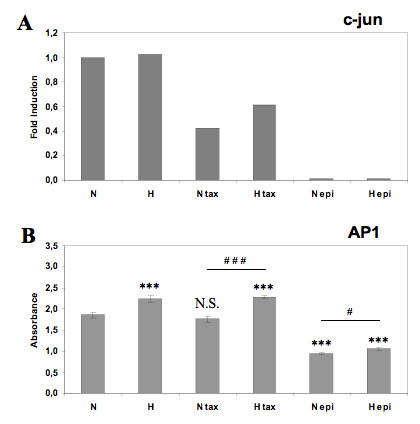
**Effect of hypoxia and/or paclitaxel or epirubicin on the c-jun mRNA expression level and AP-1 DNA binding activity**. MDA-MB-231 cells were incubated under normoxic (N) or hypoxic (H) conditions with or without paclitaxel (tax, 50 μM) or epirubicin (epi, 10 μM) for 16 hours. (A) After incubation, total RNA has been extracted and retro-transcribed in cDNA. A real time PCR has been performed with specific primers for c-jun and for RPL13A, a house-keeping gene. Results are expressed in induction level by comparison with the reference condition, normoxia. (B) After the incubation, nuclear extracts were performed and hybridized in the ELISA well containing specific DNA probes (TransAM assay). Detection was performed using an anti-c-jun antibody. Results are expressed in absorbance, as mean ± 1 SD (n = 3). N.S. = non significantly different from control, *** = significantly different from control (p < 0.001); # = significant difference between N tax and H tax (p < 0.01), ### = significant difference between N tax and H tax (p < 0.001).

The transcription factor AP-1 was also studied for its potential role in the protection effect of hypoxia against the paclitaxel-induced apoptosis. It was already shown that this factor could play a role in the hypoxia-induced anti-apoptotic effect [18]. AP-1 is composed of two proteins belonging to the jun or fos family and c-jun is the most frequent subunit. Epirubicin totally inhibited the expression of c-jun both under normoxia and hypoxia while paclitaxel decreased its expression, interestingly more under normoxia than under hypoxia (Figure 6A). The DNA binding activity of AP-1 was significantly increased under hypoxia comparatively to normoxia (Figure 6B). Epirubicin decreased AP-1 binding activity both in normoxia and hypoxia while this activity was not modified by paclitaxel. These results indicate that hypoxia was able to increase the expression of c-jun and DNA binding activity of AP-1 in the presence of paclitaxel.

**Figure 7:**
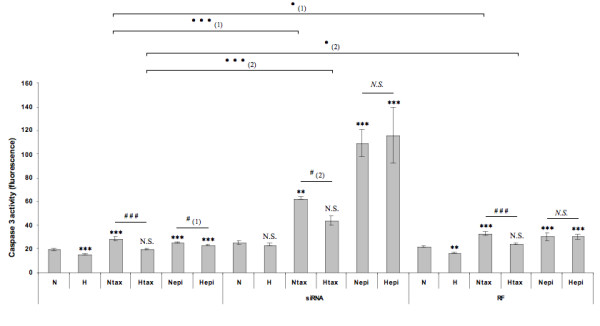
**Effect of the c-jun siRNA on the protective role of hypoxia on the paclitaxel-induced apoptosis**. MDA-MB-231 cells were incubated under normoxic (N) or hypoxic (H) conditions with or without paclitaxel (tax, 50 μM) or epirubicin (epi, 10 μM) for 16 hours, 8 h post transfection with anti-c-jun siRNA (siRNA) or RISC-free control siRNA (RF) (50 nM, 24 h). After transfection and incubation, the caspase 3 activity was assayed by measuring free AFC released from the cleavage of the caspase 3 specific substrate Ac-DEVD-AFC. Results are expressed in fluorescence intensity, as mean ± 1 SD (n = 3). Statistical analysis were determined independently for the 3 subgroups *without siRNA*, *with anti-c-jun siRNA (siRNA) *and *with RISC-free control siRNA (RF) *; N.S. = non significantly different from control (N, N siRNA or N RF), ** = significantly different from control (p < 0.01), *** = significantly different from control (p < 0.001); *N.S. *= no significant difference between N epi and H epi, ### = significant difference between N tax and H tax (p < 0.001), # = significant difference between (1) N epi and H epi or (2) N tax and H tax (p < 0.05). • = significant difference between (1) N tax and N tax RF or (2) H tax and H tax RF (p < 0.05). ••• = significant difference between (1) N tax and N tax siRNA or (2) H tax and H tax siRNA (p < 0.001),

In order to verify a possible implication of AP-1 in the protective role of hypoxia against paclitaxel-induced apoptosis, c-jun siRNA was used to inhibit the expression of this transcriptional factor. Additional file 3 shows that a concentration of 50 nM of siRNA was high enough to inhibit the synthesis and activity of c-jun while the RISC-Free control siRNA had no effect. Cells were transfected with c-jun or negative control siRNA before being incubated under normoxia or hypoxia with one or the other chemotherapeutic agent. The activity of the caspase 3 was then assessed. Specific inhibition of c-jun decreased the hypoxia-induced protection against paclitaxel-induced apoptosis (Figure 7). The negative control siRNA did not influence the caspase 3 activity measured. On the other hand, caspase 3 activity was markedly increased in c-jun siRNA transfected cells incubated under normoxia or hypoxia in the presence of paclitaxel and even more in the presence epirubicin while it was not modified in control cells. The caspase 3 activity measured for c-jun siRNA transfected cells incubated under hypoxia with paclitaxel reached a level higher than the activity measured for non transfected cells incubated under normoxia with paclitaxel.

### AP-1 protective role is associated with Mcl-1 expression modification

**Figure 8:**
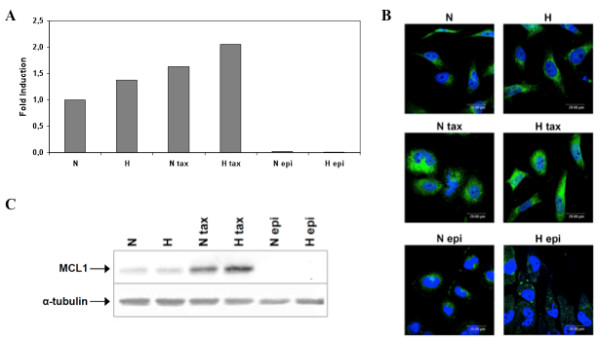
**Effect of hypoxia and/or paclitaxel or epirubicin on Mcl-1 mRNA expression level and protein expression**. MDA-MB-231 cells were incubated under normoxic (N) or hypoxic (H) conditions with or without paclitaxel (tax, 50 μM) or epirubicin (epi, 10 μM) for 16 hours (A, B). 8 h post transfection with c-jun siRNA (siRNA) or RISC-free control siRNA (RF) (50 nM, 24 h), cells were incubated under normoxic condition with or without paclitaxel (tax, 50 μM) (C). (A) After incubation, total RNA has been extracted and retro-transcribed in cDNA. A real time PCR has been performed with specific primers for Mcl-1 and for RPL13A, a house-keeping gene. Results are expressed in induction level by comparison with the reference condition, normoxia. (B) Mcl-1 was detected by immunofluorescence using specific Mcl-1 antibody. Nuclei were stained with Topro-3 (in blue). Observation was performed in semi-quantitative confocal microscopy. (C) Mcl-1 was detected in total cell extracts by western blotting, using specific antibody. α-tubulin was used to assess the total amount of proteins loaded on the gel.

**Figure 9:**
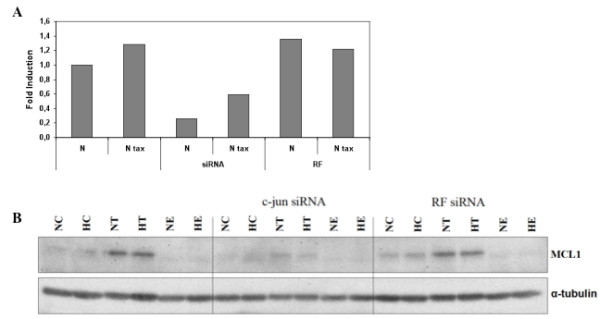
**Effect of the c-jun siRNA on Mcl-1 expression level. **(A) After transfection and incubation, total RNA has been extracted and retro-transcribed in cDNA. A real time PCR has been performed with specific primers for Mcl-1 and for RPL13A, a house-keeping gene. Results are expressed in induction level by comparison with the reference condition, normoxia. (B) Mcl-1 was detected in total cell extracts by western blotting, using specific antibody. α-tubulin was used to assess the total amount of proteins loaded on the gel.

We already point out a possible role of the anti-apoptotic protein Mcl-1 belonging to the Bcl-2 family members in the hypoxia protection against paclitaxel-induced apoptosis via the activity of HIF-1. The effect of the c-jun inhibition on Mcl-1 expression was also investigated. Hypoxia as well as paclitaxel increased Mcl-1 expression but epirubicin markedly decreased it both at the mRNA and the protein levels (Figure 8). c-jun siRNA decreased Mcl-1 mRNA expression while negative control siRNA had no effect (Figure 9A). Similar results have been observed for Mcl-1 protein abundance (Figure 9B).

All together, these results show that hypoxia was able to increase the expression of c-jun and DNA binding activity of AP-1 and that c-jun was then able to increase Mcl-1 expression which may participate to the hypoxia-induced protection against apoptosis induced by paclitaxel.

## Discussion

Breast cancer cell resistance to chemotherapy is still a great burden for patient treatment. Because of uncontrolled proliferation which arises during tumor growth, distance between tumor tissue and blood vessels capable of delivering oxygen is increased, resulting in an inadequate O_2 _supply for cells distant from the nutritive blood vessel and inducing the establishment of hypoxic regions [8,28]. It was recognized that cells undergo hypoxic conditions when the distance between blood vessels and cells exceeds the oxygen diffusion limit of 100-150 μm, [29].

Tumor hypoxia is known to enhance chemoresistance of cancer cells by a variety of ways. The delivery of drugs in hypoxic region could be counteracted because of the high distance with the blood vessels [30] and cellular uptake of many drugs could be affected because of tumor associated acidity [31,32]. The lower availability of oxygen decreases the effectiveness of drugs which require oxygen to be active [33] and compromises oxygen-dependent mechanisms such as radiotherapy [34,35]. The decrease in cancer cell proliferation induced by hypoxia and hypoxia-associated nutriment deprivation also participate to resistance against the chemotherapeutic agents which are often more effective against proliferating cells than against quiescent cells [32]. Hypoxia also induces cellular adaptations which contribute to cancer progression, such as initiation of angiogenesis and metastasis process, but also to tumor cell chemoresistance, one of these adaptations being the expression of multidrug resistance proteins such as ABC transporters. Evidence of MDR-1 or MRP1 upregulation through HIF-1 under hypoxia has been highlighted [36,37]. Our results do not show any increase in mRNA expression under hypoxia for these two proteins (results not shown). Finally, hypoxia has already been described to induce resistance to radio- and chemotherapies by modifying gene expression [14,15,38] but the exact mechanisms triggered by hypoxia that lead to this resistance are not finely characterized, neither are the genes involved.

MDA-MB-231 breast cancer cell line was used as in vitro experimental model for studying the effect of hypoxia on apoptosis induced by two commonly used chemotherapeutic agents, i.e. paclitaxel and epirubicin [39]. We observed that hypoxia differentially influenced apoptosis in these cells according to the chemotherapeutic agent. While in the presence of paclitaxel at a concentration of 50 μM during 16 hours, caspase 3 activity was inhibited under hypoxic conditions, it was not the case for epirubicin at 10 μM. Similar results were observed when DNA fragmentation was studied.

Hypoxia in tumors causes resistance to a wide variety of chemotherapeutic agents in many cell lines. Hypoxia was reported to increase resistance of human pancreatic cancer cells to gemcitabine-induced apoptosis [40], to decrease the efficacy of many drugs including cisplatin, etoposide, bleomycin, paclitaxel and mitomycin C in testicular germ cell tumors [41] or to mediate fenretinide resistance in acute lymphoblastic leukaemia cells [42].

HIF-1 being one of the main regulators of the adaptive response to hypoxia [43], its implication in the hypoxia-induced resistance to paclitaxel-induced apoptosis was investigated. Indeed, although its role as an anti- or pro-apoptotic protein is still controversy [44], many reports have evidenced an involvement of this main transcription factor activated by hypoxia in regulating the hypoxic anti-apoptotic pathway. Liu et al highlighted a contribution of HIF-1 in the hypoxia-induced chemoresistance against 5-FU, vincristine, cisplatine, etoposide and adriamycin in gastric cancer cell lines [45]. HIF-1α knockdown in breast carcinoma MCF-7 cells was shown to elevate the sensitivity to chemotherapeutic agent methotrexate [46] and resistance induced by hypoxia against doxorubicin and cisplatin in non-small cell lung cancer was reversed by silencing HIF-1α protein [47]. Down-regulation of HIF-1α by shRNA interference was also shown to reduce hypoxia-induced resistance of neuroblastoma cells against etoposide and vincristine [48]. Similarly, we show here that HIF-1α silencing reverted the hypoxia-induced protection against paclitaxel-induced apoptosis in MDA-MB-231 cells.

We hypothesized that HIF-1 could directly induce gene expression alterations that would influence the paclitaxel-induced apoptosis and that would then be responsible for the hypoxia-induced chemoresistance. From studies performed by Daniel Stiehl from the Institute of Physiology in Zurich (personal communication), HIF-2alpha is stabilized much later than HIF-1alpha in breast cancer cells: while HIF-1alpha stabilization is maximal after 4 hours, HIF-2alpha is only observed after 24 hours of hypoxia. Since the protection we observed is well before HIF-2alpha is stabilized, we though it is probably not involved.

Using TaqMan Human Apoptosis Array, expression modifications of 93 genes involved in the regulation of apoptosis were studied after HIF-1 inhibition through RNA interference. Gene expression changes were defined for each of them, in response to paclitaxel under normoxic or hypoxic conditions and with or without inhibition of HIF-1 by a siRNA approach. Gene expression patterns were then correlated with the activity of HIF-1 in order to define molecular pathway that could take part in hypoxia-induced chemoresistance against paclitaxel. HIF-1 was shown to down- or up-regulate the expression of several pro- and anti-apoptotic genes, some of them already known to be HIF-1 target. BAK, CASP3, CASP8, CASP10 and TNFRSF10A were identified as pro-apoptotic genes whose expression was decreased by HIF-1 in hypoxia when compared to normoxic conditions, in the presence or absence of paclitaxel. A decrease in BAK mRNA under hypoxia, established to be regulated through a HIF-dependent pathway, has already been observed by Sermeus et al in HepG2 cells and was supposed to play a role in the protection provided by hypoxia against etoposide-induced apoptosis [15]. TNFRSF10A (TRAIL-R1) is an apoptosis-inducing receptor for the tumor necrosis factor-related apoptosis-inducing ligand (TRAIL) which is a potent inducer of apoptosis in many cancer cells [49]. Signaling of apoptosis induced by TRAIL-R1 and its ligand involves the formation of the DISC complex, recruitment of FADD and pro-caspases 8 and 10, the activation of both caspases, followed by the activation of caspase 3 and induction of apoptosis. The consecutive Bid cleavage, mitochondrial depolarization, cytochrome c release and activation of caspase 9 could drive apoptosis through a mitochondrial pathway [50]. Correlation between the expression of the receptor and tumor progression seemed to be tumor type specific [51]. Overexpression of TRAIL-R1 was shown to efficiently killed MDA-MB-231 cells and other cancer cell lines even in the absence of exogenous TRAIL [52]. Hence, the decrease in TRAIL-R1 expression observed under hypoxia may participate to the hypoxia-induced protection.

HIF-1 was also shown to increase the expression of Mcl-1 and BNIP-3. Mcl-1 (myeloid cell leukaemia sequence 1) is an anti-apoptotic protein belonging to the Bcl-2 family. It is known to promote cell survival through suppression of cytochrome c release from mitochondria via heterodimerization and neutralization of pro-apoptotic proteins such as Bim or Bax [53]. Increased levels of Mcl-1 have been reported in a variety of human tumors, where elevated expression is associated with disease recurrence and drug resistance (for a review [54], suggesting an important role played by Mcl-1 in cancer cell survival. Previous studies also demonstrated that Mcl-1 overexpression could be regulated by HIF-1 [55] and could protect hepatoma HepG2 cells against tert-butyl hydroperoxide-induced apoptosis [14]. BNIP3, known as a proapoptotic member of the Bcl-2 family of cell death factors, was shown to be regulated by HIF-1 [56] and overexpressed in human tumors [57]. However a putative role in autophagy is more recently conferred to this protein which could thus participate to the hypoxia protection. It appears that in hypoxia, HIF-1 induces the expression of BNIP3 which triggers selective mitochondrial autophagy. This event represents an adaptive metabolic response to hypoxia to maintain redox homeostasis and to prevent cell death. [26,27].

From these results, we concluded that one putative mechanism by which hypoxia opposes to the paclitaxel-induced apoptosis could be through the regulation of the expression of some pro- and anti-apoptotic through a HIF-dependent pathway.

The transcription factor AP-1 has already been shown to be activated in cells exposed to hypoxic conditions and is known to be involved in regulation of apoptosis [58]. We then decided to investigate, in the second part of the work, the potential role of this factor in the protection effect of hypoxia against apoptosis induced by paclitaxel. AP-1 has already been shown to participate in hypoxia protection against etoposide-induced apoptosis in HepG2 cells [18]. Many publications describe cell lines in which c-jun was inhibited by various strategies, resulting in enhanced sensitivity to a panel of DNA-damaging drugs (for a review [59]. c-jun overexpression, followed by c-jun phosphorylation and AP-1 activation, in response to microtubule inhibitor vinblastine, was also shown to render MCF7 breast cancer cells markedly resistant to this drug [60].

We evidenced that hypoxia increased AP-1 DNA-binding activity and expression of c-jun in the presence of paclitaxel and that inhibition of c-jun reversed the protective effect of hypoxia against apoptosis induced by paclitaxel. c-jun was also shown to increase mRNA expression of the anti-apoptotic protein Mcl-1 which may participate to this protection.

These results suggest a potential anti-apoptotic role of AP-1 which could protect cells against paclitaxel-induced apoptosis under hypoxia and that AP-1-mediated overexpression of Mcl-1 may participate to this protection.

In conclusion, our data suggest an inhibition of the paclitaxel-induced apoptosis by hypoxia through activation of the two transcriptional factors HIF-1 and AP-1. Activation of these two factors leads to the overexpression or repression of various pro- and anti-apoptotic genes respectively. Our results provide two possible mechanisms for the induction of chemoresistance under hypoxia in MDA-MB-231breast cancer cell line. It does not exclude that hypoxia exerts its effect via other mechanisms and additional studies are now needed to evaluate the exact implication of the proteins we pointed out. Identification of proteins involved in chemoresistance is crucial in order to design new therapeutics aimed at ameliorating breast cancer patient treatment.

## Methods

### Cell culture and hypoxia incubation

Human Breast Cancer cells MDA-MB-231 were maintained in culture in 75-cm2 polystyrene flasks (Costar) with 15 ml of Roswell Park Memorial Institute medium (RPMI 1640, Invitrogen) containing 10% of fetal calf serum (Invitrogen) and incubated under an atmosphere containing 5% CO_2_.

For hypoxia experiments (1% O_2_), cells were incubated in serum-free CO_2_-independent medium (Invitrogen) supplemented with 1 mM L-glutamine (Sigma) with or without paclitaxel (Invitrogen) at 50 μM or epirubicin (Calbiochem) at 10 μM. Normoxic control cells were incubated in the same conditions but in normal atmosphere (20% O_2_).

### siRNA transfection

Silencing of HIF-1α or c-jun expression was achieved using siGENOME SMARTpool human HIF1A/c-jun from Dharmacon. RISC-free control siRNA purchased from Dharmacon was used to control for non-specific effects. For siRNA experiments, 2 × 10^6 ^cells were seeded in 75-cm^2 ^polystyrene flasks (Costar) with 10 ml of RPMI 1640 medium (RPMI) containing 10% of fetal calf serum and incubated 24 hours under an atmosphere containing 5% CO_2_. Cells were then transfected 24 hours under standard culture conditions with 50 nM siRNA using the DharmaFECT 1 (Dharmacon) transfection reagent according to the manufacturer's instructions.

The transfection media were removed and replaced by culture media for 8 hours. Cells were then trypsinized and seeded at the appropriate density in flasks or microplates, depending on the experiment to perform.

### Caspase 3 activity

The fluorogenic substrate Ac-DEVD-AFC was used to measure caspase 3 activity according to Lozano et al [61]. Cell extracts were prepared as described by Wellington et al [62]. Cells were seeded in 6-well plates (300,000 cells/well) one day before the incubation. After the incubation, the medium was recovered and centrifuged at 1,000 g for 5 min. Cells still attached to the well were scrapped in 200 μl cold PBS and recovered into a microtube. Pelleted cells were resuspended in 100 μl PBS at 4°C and also added to the microtube. The samples were centrifuged at 1,000 g for 5 min. at 4°C and the pellet resuspended in 50 μl of lysis buffer (10 mM Hepes/KOH, pH 7.0, 10% sucrose, 2 mM EDTA, 0.1% CHAPS, 5 mM dithiothreitol and 10 μg/ml aprotinin). After incubation at 4°C on a rotating wheel for 15 min, the lysates were centrifuged at 13,000 g for 5 min at 4°C and the supernatants were recovered for the assay.

The protein concentration was measured and 10 μg proteins completed to 50 μl with lysis buffer were mixed with 13 μM Ac-DEVD-AFC (BD Pharmingen) and 50 μl reaction buffer (40 mM PIPES, pH 7.2, 200 mM NaCl, 2 mM EDTA, 0.2% CHAPS, 0.10% sucrose and 10 mM dithiothreitol). The reaction was allowed to take place for 1 hour at 37°C and the fluorescence generated by the release of the fluorogenic group AFC on cleavage by caspase 3 was measured by excitation at 400 nm and emission at 505 nm.

### DNA fragmentation

The measurement of cytoplasmic histone-associated DNA fragments (mono- and oligonucleosomes) after induction of cell death was performed with the «cell death detection ELISA» (Roche Molecular Biochemicals). After treatment, cells were lysed with the incubation buffer and the cytoplasmic fraction recovered. ELISA was performed according to the manufacturer's protocol.

### Immunofluorescence staining and confocal microscopy

MDA-MB-231 cells were seeded at 30,000 cells/well in 24-well plates and 24 hours later were incubated for 16 hours with or without paclitaxel or epirubicin under normoxic or hypoxic conditions. Immunofluorescence staining was performed as described in [18].

Primary antibody for Bim staining was rabbit anti-Bim (# 2819 Cell Signaling) (1/100 dilution). Primary antibody for Mcl1 staining was rabbit anti-Mcl1 (# 819 Santa Cruz) (1/100 dilution). Alexa Fluor-488-conjugated anti rabbit IgG antibody (Molecular Probes) was used at 1/1,000 dilution. Primary antibody for α-tubulin staining was mouse anti-α-tubulin (# T5186 Sigma) (1/100 dilution). Alexa Fluor-546-conjugated anti-mouse IgG antibody (Molecular Probes) was used at 1/1,000 dilution.

### Western blotting

Cells, seeded and incubated in 25 cm2 flasks, were scrapped in 200 μl of lysis buffer (Tris 40 mM pH 7.5, KCl 150 mM, EDTA 1 mM, triton X-100 1%) containing a protease inhibitor mixture («Complete» from Roche Molecular Biochemicals, 1 tablet in 2 ml H_2_O, added at a 1/25 dilution) and phosphatase inhibitors (NaVO_3 _25 mM, p-Nitrophenyl Phosphate (PNPP) 250 mM, β-glycerophosphate 250 mM and NaF 125 mM, at a 1/25 dilution). Western blot analysis was performed as described in [18] using mouse anti-HIF-1α antibody (# 610958 Becton Dickinson) used at 1/1,500 dilution or rabbit anti-MCL1 antibody (# 4572 Cell Signaling) used at 1/5000 dilution. Mouse anti-β-actin antibody (A5441 Sigma) (final dilution 1/100,000) and mouse anti-α-tubulin (Sigma) (final dilution 1/3000) were used for normalization. Mouse and rabbit IgG horseradish peroxidase-linked antibody (Amersham Pharmacia Biotech) was used at 1/300,000 dilution as secondary antibodies.

### Nuclear protein extraction

Nuclear protein extractions in high salt buffer were prepared as previously described [63]. Briefly, cells seeded in 75 cm2 flasks (Corning) were incubated with or without paclitaxel at 50 μM or epirubicin at 10 μM under normoxic or hypoxic conditions for 16 hours. At the end of the incubation, cells were rinsed with PBS containing 1 mM Na_2_MoO_4 _and 5 mM NaF. They were then incubated on ice for 3 minutes with 10 ml cold Hypotonic Buffer (HB 1X, 20 mM HEPES, 5 mM NaF, 1 mM Na_2_MoO_4_, 0.1 mM EDTA) and harvested in 500 μl HB containing 0.5% NP-40 (Sigma). Cell lysates were centrifuged 1 minute at 13,000 rpm and sedimented nuclei were resuspended in 50 μl HB containing 20% glycerol and a protease inhibitor cocktail (Roche) and phosphatase inhibitors (1 mM Na_3_VO_4_, 5 mM NaF, 10 mM p-nitrophenylphosphate, 10 mM β-glycerophosphate). Extraction was performed for 30 minutes at 4°C by the addition of 50 μl HB containing 20% glycerol, 0.8 M NaCl and protease/phosphatase inhibitors. Nuclear extracts from the supernatant were recovered after centrifugation for 10 minutes at 13,000 rpm.

### DNA-binding assay

DNA-binding assays using TransAM ELISA kit (Active Motif) for detecting transcription factor DNA binding activity was performed according to the manufacturer's recommendations. Briefly, 5 μg of nuclear proteins were incubated for 1 hour in a 96-well plate coated with a double-stranded oligonucleotide containing the consensus sequence recognized by the transcription factor to be assayed. The transcription factor bound to DNA was detected using a specific primary antibody (rabbit anti-c-Jun (SC-1964 Santa Cruz), mouse anti-HIF-1α (H72320 Transduction Laboratories)). Colorimetric reaction was then performed with a HRP-conjugated anti-rabbit or anti-mouse IgG antibody and absorbance was measured at 450 nm in a spectrophotometer.

### Transient transfection and luciferase assay

MDA-MB-231 transfections were performed in 24-well plates (35,000 cells per well) with SuperFect reagent (Qiagen). 1384 ng of the reporter plasmid pGL3(PGK-HRE6)-tk-Luc containing binding sites for HIF-1 upstream of the firefly luciferase gene [64] were co-transfected with 115.4 ng of normalization vector (pCMVβ vector coding for the β-galactosidase, Promega) in RMPI without serum for 5 hours.

The transfection media were removed and replaced by culture media for 24 hours. Cells were then incubated under hypoxia for 16 hours. After hypoxia incubation, β-galactosidase was assayed in parallel to the firefly luciferase activity, assayed in a luminometer using the Luciferase Reporter Assay System (Promega). Results are expressed as means of the ratio between the firefly luciferase activity and the β-galactosidase activity.

### Real time RT-PCR

After the incubation, total RNA was extracted using the Total RNAgent extraction kit (Promega). mRNA contained in 2 μg total RNA was reverse transcribed using SuperScript II Reverse Transcriptase (InVitrogen) and oligodT primers according to the manufacturer's instructions. Forward and reverse primers for RPL13A, C-JUN, MCL-1, LDHA, BNIP3 and TNFRSF1B were designed using the Primer Express 1.5 software (Applied Biosystems). Amplification reaction assays contained 1× SYBR Green PCR Mastermix (Applied Biosystems) and primers (Applied Biosystems) at the optimal concentrations. A hot start at 95°C for 5 minutes was followed by 40 cycles at 95°C for 15 seconds and 65°C for 1 minute using an ABI PRISM 7000 SDS thermal cycler (Applied Biosystems). RPL13A was used as the reference gene for normalization and mRNA expression level was quantified using the threshold cycle method.

### Taqman Low Density Array

After the incubation, total RNA was extracted using the Total RNAgent extraction kit (Promega). mRNA contained in 2 μg total RNA was reverse transcribed using the "High Capacity cDNA Archive" kit from Applied Biosystems according to the manufacturer's instructions. 100 ng of retrotranscribed total RNA in 50 μl were then mixed with 50 μl of the "Taqman Universal PCR master Mix" (Applied Biosystems) and loaded into one of the 8 fill ports the microfluidic array. "TaqMan Human Apoptosis Arrays" are 384-well micro fluidic cards that contain assays for 93 human genes in addition to 3 endogenous controls. They enable to perform 96 real-time PCR reactions simultaneously for 4 samples, allowing the detection of 96 genes. mRNA expression level was quantified using the threshold cycle method with 18S as the reference gene.

### Statistics

SigmaStat software (Jandle Scientific, Germany) was used for the statistical analysis. Data are presented as means ± SD and were evaluated by one-way ANOVA, using the Holm-Sidak method.

## Competing interests

The authors declare that they have no competing interests.

## Authors' contributions

LF, AN carried out all the experiments, NN carried the immunofluorescence studies, MR participated in the design of the study, CM conceived the study, participated in its design and coordination and helped to draft the manuscript. All authors read and approved the final manuscript

## Supplementary Material

Additional file 1 **Effect of HIF-1α silencing on HIF-1α, Bnip3 and HIF-2α expression**. (A) MDA-MB-231 cells were transfected 24 h with anti-HIF-1α siRNA (siRNA) or RISC-free control siRNA (RF) (50 nM). The transfection media were removed and replaced by culture media for 24 or 48 hours. Cells were then incubated under normoxic (N) or hypoxic (H) conditions for 16 hours. After transfection and incubation, HIF-1α was detected in total cell extracts by western blotting, using specific antibody. α-tubulin was used to assess the total amount of proteins loaded on the gel. (B) MDA-MB-231 cells were transfected 24 h with anti-HIF-1α siRNA (siRNA) or RISC-free control siRNA (RF) (50 nM). The transfection media were removed and replaced by culture media for 24. Cells were then incubated under hypoxic (H) conditions for 16 hours. After transfection and incubation, total RNA has been extracted and retro-transcribed in cDNA. A real time PCR has been performed with specific primers for HIF-1α, Bnip3, HIF-2α and for RPL13A, a house-keeping gene. Results are expressed in induction level by comparison with the reference condition, hypoxia. Click here for file

Additional file 2 **Gene expression profiling, for genes involved in regulating apoptosis**. MDA-MB-231 cells were transfected or not with HIF-1α siRNA (siRNA) or RISC-free control siRNA (RF) (50 nM, 24 h) and were incubated with or without paclitaxel (tax) under normoxic (N) or hypoxic (H) conditions. TaqMan Human Apoptosis Array (Applied Biosystems) was used to perform real time PCR for 93 human genes implicated in apoptosis in addition to three endogenous controls. 18S was used as the house-keeping gene for data normalization. Results obtained for the 77 genes detected after real time PCR reactions are expressed in induction level by comparison with the reference condition, normoxia (RQ) and cycle threshold (Ct) are given. Click here for file

Additional file 3 **Effect of c-jun silencing on c-jun expression**. MDA-MB-231 cells were transfected 24 h with c-jun siRNA (siRNA) or RISC-free control siRNA (RF) (50 nM). Cells were then incubated with or without paclitaxel (tax, 50 μM) under normoxic (N) conditions for 16 hours. After transfection and incubation, total RNA has been extracted and retro-transcribed in cDNA. A real time PCR has been performed with specific primers for C-JUN and for RPL13A, a house-keeping gene. Results are expressed in induction level by comparison with the reference condition, normoxia. Click here for file
