# Supplementary material for: Anti-apoptotic role of HIF-1 and AP-1 in paclitaxel exposed breast cancer cells under hypoxia
Source: Mol Cancer. 2010 Jul 13;9:191. doi: 10.1186/1476-4598-9-191 (PMC3098009; doi:10.1186/1476-4598-9-191)
Supplement: Additional file 1 — Effect of HIF-1α silencing on HIF-1α, Bnip3 and HIF-2α expression. (A) MDA-MB-231 cells were transfected 24 h with anti-HIF-1α siRNA (siRNA) or RISC-free control siRNA (RF) (50 nM). The transfection media were removed and replaced by culture media for 24 or 48 hours. Cells were then incubated under normoxic (N) or hypoxic (H) conditions for 16 hours. After transfection and incubation, HIF-1α was detected in total cell extracts by western blotting, using specific antibody. α-tubulin was used to assess the total amount of proteins loaded on the gel. (B) MDA-MB-231 cells were transfected 24 h with anti-HIF-1α siRNA (siRNA) or RISC-free control siRNA (RF) (50 nM). The transfection media were removed and replaced by culture media for 24. Cells were then incubated under hypoxic (H) conditions for 16 hours. After transfection and incubation, total RNA has been extracted and retro-transcribed in cDNA. A real time PCR has been performed with specific primers for HIF-1α, Bnip3, HIF-2α and for RPL13A, a house-keeping gene. Results are expressed in induction level by comparison with the reference condition, hypoxia. [file 1476-4598-9-191-S1.PDF]

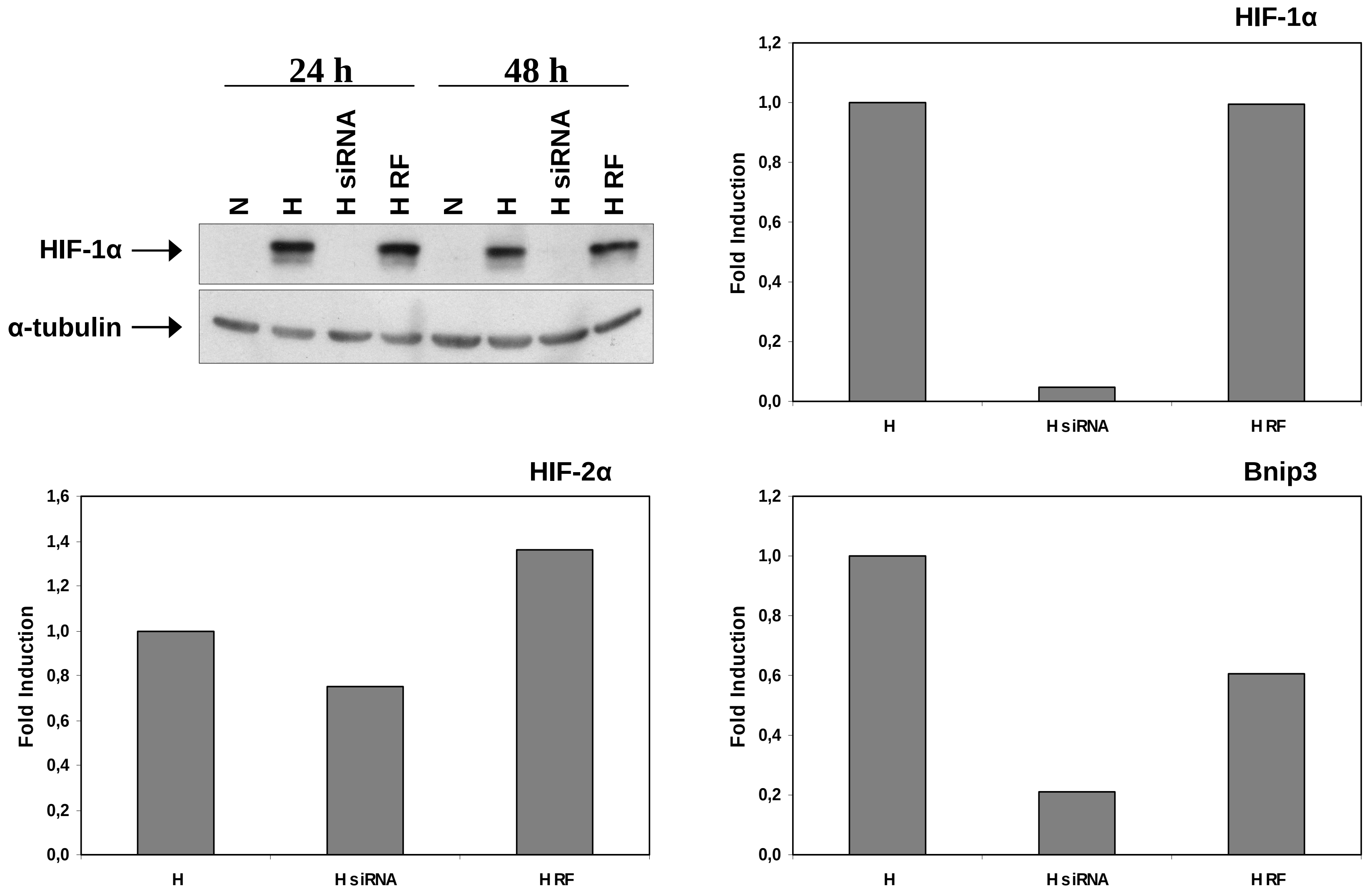

Additional file 1

Effect of HIF-1α silencing on HIF-1α, Bnip3 and HIF-2α expression. (A) MDA-MB-231 cells were transfected 24 h with anti-HIF-1α siRNA (siRNA) or RISC-free control siRNA (RF) (50nM). The transfection media were removed and replaced by culture media for 24 or 48 hours. Cells were then incubated under normoxic (N) or hypoxic (H) conditions for 16 hours. After transfection and incubation, HIF-1α was detected in total cell extracts by western blotting, using specific antibody. α-tubulin was used to assess the total amount of proteins loaded on the gel. (B) MDA-MB-231 cells were transfected 24 h with anti-HIF-1α siRNA (siRNA) or RISC-free control siRNA (RF) (50nM). The transfection media were removed and replaced by culture media for 24. Cells were then incubated under hypoxic (H) conditions for 16 hours. After transfection and incubation, total RNA has been extracted and retro-transcripted in cDNA. A real time PCR has been performed with specific primers for HIF-1α, Bnip3, HIF-2α and for RPL13A, a house-keeping gene. Results are expressed in induction level by comparison with the reference condition, hypoxia.
