# Supplementary material for: Anti-apoptotic role of HIF-1 and AP-1 in paclitaxel exposed breast cancer cells under hypoxia
Source: Mol Cancer. 2010 Jul 13;9:191. doi: 10.1186/1476-4598-9-191 (PMC3098009; doi:10.1186/1476-4598-9-191)
Supplement: Additional file 2 — Gene expression profiling, for genes involved in regulating apoptosis. MDA-MB-231 cells were transfected or not with HIF-1α siRNA (siRNA) or RISC-free control siRNA (RF) (50 nM, 24 h) and were incubated with or without paclitaxel (tax) under normoxic (N) or hypoxic (H) conditions. TaqMan Human Apoptosis Array (Applied Biosystems) was used to perform real time PCR for 93 human genes implicated in apoptosis in addition to three endogenous controls. 18S was used as the house-keeping gene for data normalization. Results obtained for the 77 genes detected after real time PCR reactions are expressed in induction level by comparison with the reference condition, normoxia (RQ) and cycle threshold (Ct) are given. [file 1476-4598-9-191-S2.PDF]

Additional file 2

Gene expression profiling, for genes involved in regulating apoptosis, in MDA-MB-231 cells transfected or not with HIF-1α siRNA (siRNA) or RISC-free control siRNA (RF) (50nM, 24h), incubated with or without paclitaxel (tax) under normoxic (N) or hypoxic (H) conditions. TaqMan Human Apoptosis Array (Applied Biosystems) was used to perform real time PCR for 93 human genes implicated in apoptosis in addition to three endogenous controls. 18S was used as the house-keeping gene for data normalization. Results obtained for the 77 genes detected after real time PCR reactions are expressed in induction level by comparison with the reference condition, normoxia (RQ) and cycle threshold (Ct) are given.

APAF1					siRNA				RF			
	N	H	N tax	H tax	N	H	N tax	H tax	N	H	N tax	H tax
Ct	28,2	27,6	27,9	27,7	27,3	27,2	27,6	28,0	27,8	27,2	28,5	27,3
RQ	1,00	1,04	0,74	1,13	1,34	1,27	0,62	0,68	0,92	1,26	0,49	1,04

BAD					siRNA				RF			
	N	H	N tax	H tax	N	H	N tax	H tax	N	H	N tax	H tax
Ct	28,3	28,7	28,9	29,1	28,3	28,4	27,8	28,8	28,4	28,4	28,7	29,0
RQ	1,00	0,53	0,42	0,47	0,76	0,63	0,63	0,41	0,69	0,61	0,48	0,37

BAK1					siRNA				RF			
	N	H	N tax	H tax	N	H	N tax	H tax	N	H	N tax	H tax
Ct	27,4	28,0	26,7	27,6	26,9	27,2	26,7	27,0	27,1	27,6	26,8	27,3
RQ	1,00	0,47	1,01	0,69	1,10	0,73	0,74	0,81	0,91	0,58	0,97	0,63

BAX					siRNA				RF			
	N	H	N tax	H tax	N	H	N tax	H tax	N	H	N tax	H tax
Ct	25,5	25,9	25,2	26,0	25,0	25,4	25,1	25,4	25,0	25,5	25,3	25,5
RQ	1,00	0,54	0,78	0,58	1,05	0,67	0,59	0,62	0,99	0,65	0,69	0,60

BBC3					siRNA				RF			
	N	H	N tax	H tax	N	H	N tax	H tax	N	H	N tax	H tax
Ct	29,9	29,1	28,6	27,9	29,3	28,3	27,9	27,8	29,3	29,0	28,3	27,9
RQ	1,00	1,21	1,58	3,37	1,18	2,06	1,86	2,58	1,10	1,30	1,97	2,38

BCAP31					siRNA				RF			
	N	H	N tax	H tax	N	H	N tax	H tax	N	H	N tax	H tax
Ct	24,1	23,9	23,8	24,2	23,5	24,0	23,6	24,1	23,9	23,7	23,9	23,8
RQ	1,00	0,81	0,74	0,77	1,09	0,70	0,62	0,60	0,85	0,85	0,72	0,72

BCL10					siRNA				RF			
	N	H	N tax	H tax	N	H	N tax	H tax	N	H	N tax	H tax
Ct	26,5	25,7	25,9	26,2	26,4	26,7	25,5	26,0	26,3	26,0	26,1	25,0
RQ	1,00	1,22	0,98	1,04	0,83	0,59	0,93	0,84	0,86	0,94	0,84	1,76

BCL2					siRNA				RF			
	N	H	N tax	H tax	N	H	N tax	H tax	N	H	N tax	H tax
Ct	31,0	33,1	31,2	32,6	30,6	31,0	31,5	32,3	30,8	31,8	31,5	32,1
RQ	1,00	0,16	0,52	0,25	0,97	0,64	0,30	0,24	0,81	0,36	0,42	0,27

BCL2L11					siRNA				RF			
	N	H	N tax	H tax	N	H	N tax	H tax	N	H	N tax	H tax
Ct	29,0	29,0	28,1	28,6	28,3	29,2	28,1	27,9	28,6	29,3	28,3	27,7
RQ	1,00	0,66	1,11	1,07	1,16	0,56	0,80	1,18	0,94	0,52	0,94	1,38

BCL2L13					siRNA				RF			
	N	H	N tax	H tax	N	H	N tax	H tax	N	H	N tax	H tax
Ct	29,4	30,0	28,4	29,1	29,1	28,9	28,7	28,9	29,4	29,0	28,9	28,9
RQ	1,00	0,45	1,24	1,03	0,96	0,95	0,72	0,82	0,73	0,86	0,89	0,82

BCL2L1					siRNA				RF			
	N	H	N tax	H tax	N	H	N tax	H tax	N	H	N tax	H tax
Ct	24,7	25,8	25,2	26,0	25,7	26,4	25,5	26,6	24,7	25,4	26,1	25,6
RQ	1,00	0,32	0,42	0,33	0,38	0,20	0,24	0,15	0,72	0,41	0,23	0,31

BCL2L2					siRNA				RF			
	N	H	N tax	H tax	N	H	N tax	H tax	N	H	N tax	H tax
Ct	27,4	28,1	26,1	27,1	27,1	27,5	26,0	27,0	27,2	27,7	26,1	26,5
RQ	1,00	0,42	1,59	1,03	0,93	0,63	1,20	0,79	0,85	0,55	1,52	1,09

BCL3					siRNA				RF			
	N	H	N tax	H tax	N	H	N tax	H tax	N	H	N tax	H tax
Ct	26,3	26,8	27,3	28,0	25,7	25,4	26,9	26,8	26,1	26,3	27,3	27,4
RQ	1,00	0,49	0,29	0,24	1,10	1,14	0,29	0,40	0,80	0,63	0,29	0,26

BID					siRNA				RF			
	N	H	N tax	H tax	N	H	N tax	H tax	N	H	N tax	H tax
Ct	27,9	27,7	28,9	29,5	27,8	28,0	28,8	28,9	27,6	27,4	28,8	28,5
RQ	1,00	0,83	0,32	0,27	0,81	0,64	0,24	0,29	0,90	0,98	0,35	0,40

BIK					siRNA				RF			
	N	H	N tax	H tax	N	H	N tax	H tax	N	H	N tax	H tax
Ct	32,8	32,9	31,0	30,9	31,6	31,3	30,9	30,5	32,0	31,9	31,0	30,1
RQ	1,00	0,66	2,12	2,94	1,76	1,85	1,67	2,81	1,29	1,25	2,19	3,75

BIRC1					siRNA				RF			
	N	H	N tax	H tax	N	H	N tax	H tax	N	H	N tax	H tax
Ct	31,9	30,8	30,6	32,0	30,8	31,5	30,7	31,9	30,9	31,3	30,9	30,1
RQ	1,00	1,45	1,45	0,73	1,54	0,84	1,01	0,57	1,39	0,98	1,23	2,01

BIRC2					siRNA				RF			
	N	H	N tax	H tax	N	H	N tax	H tax	N	H	N tax	H tax
Ct	27,1	26,4	25,4	26,3	26,6	26,4	26,0	26,1	25,9	25,7	26,8	25,4
RQ	1,00	1,11	1,97	1,41	1,04	1,05	0,94	1,20	1,59	1,70	0,77	1,87

BIRC3					siRNA				RF			
	N	H	N tax	H tax	N	H	N tax	H tax	N	H	N tax	H tax
Ct	30,6	30,6	29,3	29,8	28,6	28,8	28,5	29,0	29,2	29,3	29,9	29,1
RQ	1,00	0,74	1,58	1,47	2,98	2,29	1,99	1,85	1,96	1,69	1,02	1,67

BIRC4					siRNA				RF			
	N	H	N tax	H tax	N	H	N tax	H tax	N	H	N tax	H tax
Ct	27,9	28,3	27,1	27,9	27,9	27,9	28,0	28,1	27,6	27,7	27,9	27,6
RQ	1,00	0,53	1,06	0,84	0,72	0,65	0,41	0,50	0,88	0,75	0,62	0,72

DEDD2					siRNA				RF			
	N	H	N tax	H tax	N	H	N tax	H tax	N	H	N tax	H tax
Ct	28,6	28,2	27,5	27,6	28,5	28,5	27,0	27,2	28,5	28,0	27,9	27,3
RQ	1,00	0,98	1,38	1,63	0,82	0,74	1,33	1,55	0,78	1,05	1,07	1,50

DEDD					siRNA				RF			
	N	H	N tax	H tax	N	H	N tax	H tax	N	H	N tax	H tax
Ct	26,6	26,9	26,2	26,9	26,9	27,4	26,9	27,3	26,0	26,8	26,6	26,5
RQ	1,00	0,55	0,82	0,64	0,59	0,38	0,37	0,35	1,07	0,59	0,62	0,61

DIABLO					siRNA				RF			
	N	H	N tax	H tax	N	H	N tax	H tax	N	H	N tax	H tax
Ct	25,6	25,3	25,4	25,4	25,0	25,0	24,7	25,3	25,1	25,4	25,6	25,0
RQ	1,00	0,84	0,69	0,88	1,10	0,98	0,79	0,70	0,98	0,75	0,62	0,84

ESRRBL1					siRNA				RF			
	N	H	N tax	H tax	N	H	N tax	H tax	N	H	N tax	H tax
Ct	28,1	28,7	28,2	29,2	27,8	27,8	28,5	28,9	28,2	27,8	28,9	28,9
RQ	1,00	0,47	0,59	0,39	0,95	0,80	0,33	0,33	0,68	0,80	0,35	0,33

FADD					siRNA				RF			
	N	H	N tax	H tax	N	H	N tax	H tax	N	H	N tax	H tax
Ct	28,0	28,8	28,3	28,9	28,0	28,3	27,6	28,4	28,1	28,6	28,2	28,7
RQ	1,00	0,41	0,53	0,43	0,76	0,54	0,58	0,47	0,70	0,45	0,55	0,38

FAS					siRNA				RF			
	N	H	N tax	H tax	N	H	N tax	H tax	N	H	N tax	H tax
Ct	28,9	28,8	27,2	28,1	27,7	28,3	27,1	27,7	28,7	28,3	28,0	27,9
RQ	1,00	0,76	1,98	1,42	1,68	0,98	1,54	1,37	0,84	0,99	1,19	1,19

GAPDH					siRNA				RF			
	N	H	N tax	H tax	N	H	N tax	H tax	N	H	N tax	H tax
Ct	20,0	18,1	19,5	19,0	19,4	19,7	19,1	19,6	19,4	18,3	19,8	18,3
RQ	1,00	2,47	0,87	1,60	1,12	0,79	0,81	0,74	1,08	2,10	0,72	1,91

HIP1					siRNA				RF			
	N	H	N tax	H tax	N	H	N tax	H tax	N	H	N tax	H tax
Ct	28,6	29,2	29,2	30,0	29,4	28,9	30,2	30,1	28,2	28,7	29,8	29,4
RQ	1,00	0,44	0,40	0,31	0,42	0,53	0,14	0,20	0,94	0,59	0,26	0,34

HTRA2					siRNA				RF			
	N	H	N tax	H tax	N	H	N tax	H tax	N	H	N tax	H tax
Ct	28,0	29,1	27,5	28,4	27,8	28,2	27,8	28,4	28,0	28,4	27,8	28,1
RQ	1,00	0,31	0,86	0,61	0,87	0,55	0,51	0,44	0,74	0,50	0,68	0,52

IKBKB					siRNA				RF			
	N	H	N tax	H tax	N	H	N tax	H tax	N	H	N tax	H tax
Ct	27,2	27,8	26,4	27,6	27,3	27,3	27,3	27,4	26,9	27,2	27,0	27,1
RQ	1,00	0,47	1,06	0,62	0,72	0,61	0,42	0,52	0,88	0,66	0,71	0,62

IKBKE					siRNA				RF			
	N	H	N tax	H tax	N	H	N tax	H tax	N	H	N tax	H tax
Ct	28,8	29,1	28,6	29,1	28,5	29,1	28,8	28,9	28,1	28,7	29,0	28,8
RQ	1,00	0,56	0,69	0,64	0,91	0,52	0,45	0,56	1,17	0,68	0,53	0,59

IKBKG					siRNA				RF			
	N	H	N tax	H tax	N	H	N tax	H tax	N	H	N tax	H tax
Ct	27,1	27,1	26,4	27,1	26,8	26,9	26,1	26,6	26,5	26,5	26,2	26,2
RQ	1,00	0,68	0,96	0,81	0,93	0,73	0,88	0,83	1,07	1,00	1,10	1,05

LRDD					siRNA				RF			
	N	H	N tax	H tax	N	H	N tax	H tax	N	H	N tax	H tax
Ct	29,7	30,5	30,0	30,9	29,3	30,0	29,8	30,2	28,8	29,8	31,0	30,4
RQ	1,00	0,40	0,51	0,35	0,97	0,52	0,42	0,41	1,34	0,63	0,25	0,37

MCL1					siRNA				RF			
	N	H	N tax	H tax	N	H	N tax	H tax	N	H	N tax	H tax
Ct	25,0	24,1	23,6	23,6	24,8	24,8	24,2	24,4	24,5	24,0	24,6	23,3
RQ	1,00	1,24	1,54	2,09	0,86	0,70	0,74	0,88	0,97	1,29	0,80	1,83

NALP1					siRNA				RF			
	N	H	N tax	H tax	N	H	N tax	H tax	N	H	N tax	H tax
Ct	30,6	30,0	29,2	30,2	30,8	30,8	29,1	29,9	29,8	30,2	29,8	29,5
RQ	1,00	1,09	1,65	1,07	0,66	0,57	1,23	0,98	1,29	0,89	1,11	1,27

NFKB1					siRNA				RF			
	N	H	N tax	H tax	N	H	N tax	H tax	N	H	N tax	H tax
Ct	27,8	28,0	26,9	28,0	27,2	27,5	27,2	27,3	27,3	27,8	27,9	27,4
RQ	1,00	0,61	1,15	0,71	1,15	0,81	0,67	0,86	1,02	0,67	0,58	0,76

NFKB2					siRNA				RF			
	N	H	N tax	H tax	N	H	N tax	H tax	N	H	N tax	H tax
Ct	26,2	26,1	24,4	24,9	25,5	25,2	24,3	24,1	25,5	25,8	24,6	24,5
RQ	1,00	0,78	2,20	1,98	1,26	1,31	1,62	2,50	1,19	0,91	1,86	1,92

NFKBIA					siRNA				RF			
	N	H	N tax	H tax	N	H	N tax	H tax	N	H	N tax	H tax
Ct	25,8	26,3	25,8	26,4	24,9	25,0	25,4	25,4	25,1	25,4	26,0	25,4
RQ	1,00	0,47	0,58	0,51	1,36	1,08	0,54	0,74	1,15	0,82	0,53	0,74

NFKBIB					siRNA				RF			
	N	H	N tax	H tax	N	H	N tax	H tax	N	H	N tax	H tax
Ct	28,8	29,3	27,5	28,6	28,2	28,5	27,5	28,0	28,0	29,0	27,9	28,0
RQ	1,00	0,51	1,54	0,98	1,17	0,84	1,11	1,04	1,25	0,59	1,17	1,05

BIRC5					siRNA				RF			
	N	H	N tax	H tax	N	H	N tax	H tax	N	H	N tax	H tax
Ct	25,0	25,6	24,6	25,3	24,6	25,2	24,8	24,9	24,7	25,2	24,9	24,9
RQ	1,00	0,44	0,82	0,66	1,01	0,55	0,51	0,62	0,87	0,59	0,67	0,60

BIRC6					siRNA				RF			
	N	H	N tax	H tax	N	H	N tax	H tax	N	H	N tax	H tax
Ct	27,4	27,8	27,1	28,1	27,2	27,5	27,0	27,9	27,2	27,3	27,9	27,6
RQ	1,00	0,50	0,73	0,49	0,85	0,59	0,57	0,40	0,82	0,68	0,42	0,49

BIRC8					siRNA				RF			
	N	H	N tax	H tax	N	H	N tax	H tax	N	H	N tax	H tax
Ct	32,0	33,1	31,3	32,3	31,7	31,9	31,3	31,6	32,4	31,8	31,8	31,5
RQ	1,00	0,33	0,99	0,66	0,94	0,70	0,68	0,76	0,54	0,76	0,69	0,82

BNIP3					siRNA				RF			
	N	H	N tax	H tax	N	H	N tax	H tax	N	H	N tax	H tax
Ct	26,2	22,4	25,2	22,6	25,9	26,0	25,6	26,0	25,6	22,3	26,0	22,1
RQ	1,00	9,70	1,22	9,64	0,91	0,74	0,68	0,66	1,10	10,12	0,74	9,96

BNIP3L					siRNA				RF			
	N	H	N tax	H tax	N	H	N tax	H tax	N	H	N tax	H tax
Ct	25,9	24,0	25,7	24,8	26,2	25,6	26,1	26,2	25,9	23,7	26,2	24,4
RQ	1,00	2,57	0,73	1,75	0,61	0,82	0,38	0,47	0,74	3,11	0,51	1,68

BOK					siRNA				RF			
	N	H	N tax	H tax	N	H	N tax	H tax	N	H	N tax	H tax
Ct	28,0	28,1	27,6	28,5	27,8	27,9	27,3	28,4	27,9	28,0	27,6	27,7
RQ	1,00	0,65	0,78	0,58	0,85	0,69	0,72	0,43	0,75	0,65	0,79	0,71

CARD4					siRNA				RF			
	N	H	N tax	H tax	N	H	N tax	H tax	N	H	N tax	H tax
Ct	30,7	32,0	30,2	31,7	29,8	30,6	30,0	30,9	29,4	31,5	30,5	31,0
RQ	1,00	0,28	0,90	0,40	1,38	0,68	0,74	0,51	1,83	0,39	0,71	0,48

CARD6					siRNA				RF			
	N	H	N tax	H tax	N	H	N tax	H tax	N	H	N tax	H tax
Ct	28,7	31,5	29,6	30,4	28,8	30,6	27,9	30,1	28,1	16,1	30,0	27,8
RQ	1,00	0,10	0,33	0,25	0,69	0,18	0,77	0,22	1,14	####	0,26	1,12

CASP10					siRNA				RF			
	N	H	N tax	H tax	N	H	N tax	H tax	N	H	N tax	H tax
Ct	30,6	31,1	29,2	30,6	29,7	30,4	29,5	30,0	29,5	30,5	30,0	30,4
RQ	1,00	0,48	1,59	0,80	1,36	0,75	0,93	0,92	1,52	0,72	0,94	0,66

CASP2					siRNA				RF			
	N	H	N tax	H tax	N	H	N tax	H tax	N	H	N tax	H tax
Ct	25,9	26,9	26,3	27,4	25,6	26,0	26,1	26,6	25,5	26,2	27,0	26,9
RQ	1,00	0,35	0,49	0,29	0,95	0,63	0,39	0,36	0,94	0,54	0,30	0,29

CASP3					siRNA				RF			
	N	H	N tax	H tax	N	H	N tax	H tax	N	H	N tax	H tax
Ct	26,7	27,6	26,2	27,0	26,0	26,2	25,8	26,4	26,6	27,3	26,9	26,6
RQ	1,00	0,39	0,91	0,67	1,24	0,95	0,83	0,73	0,82	0,44	0,56	0,65

CASP4					siRNA				RF			
	N	H	N tax	H tax	N	H	N tax	H tax	N	H	N tax	H tax
Ct	29,2	29,9	27,1	28,4	29,2	29,8	27,7	28,5	28,5	29,0	27,9	28,0
RQ	1,00	0,43	2,60	1,37	0,72	0,43	1,23	0,97	1,13	0,76	1,51	1,37

CASP6					siRNA				RF			
	N	H	N tax	H tax	N	H	N tax	H tax	N	H	N tax	H tax
Ct	27,0	27,8	26,9	28,4	26,4	26,9	26,8	27,5	26,3	27,6	27,9	27,6
RQ	1,00	0,39	0,62	0,29	1,11	0,68	0,50	0,39	1,16	0,41	0,31	0,37

CASP7					siRNA				RF			
	N	H	N tax	H tax	N	H	N tax	H tax	N	H	N tax	H tax
Ct	28,0	28,4	27,4	28,4	28,3	28,6	28,1	28,6	27,5	27,9	27,9	27,9
RQ	1,00	0,50	0,93	0,58	0,57	0,43	0,40	0,39	0,97	0,71	0,66	0,59

CASP8AP2					siRNA				RF			
	N	H	N tax	H tax	N	H	N tax	H tax	N	H	N tax	H tax
Ct	29,4	30,1	29,7	30,7	29,4	29,9	30,3	30,8	29,2	29,7	30,8	30,6
RQ	1,00	0,41	0,49	0,32	0,72	0,44	0,24	0,21	0,81	0,50	0,24	0,25

CASP8					siRNA				RF			
	N	H	N tax	H tax	N	H	N tax	H tax	N	H	N tax	H tax
Ct	27,9	28,4	27,6	28,7	27,7	27,8	27,8	27,9	27,7	28,0	28,2	28,2
RQ	1,00	0,47	0,76	0,47	0,86	0,67	0,46	0,59	0,84	0,62	0,51	0,46

CASP9					siRNA				RF			
	N	H	N tax	H tax	N	H	N tax	H tax	N	H	N tax	H tax
Ct	25,5	26,0	25,6	25,6	25,0	25,8	25,5	25,7	etermi	26,0	25,9	25,8
RQ	1,00	0,49	0,55	0,76	1,01	0,52	0,44	0,51	0,00	0,47	0,45	0,48

CFLAR					siRNA				RF			
	N	H	N tax	H tax	N	H	N tax	H tax	N	H	N tax	H tax
Ct	28,4	29,0	27,8	28,5	28,3	28,9	28,5	29,0	27,8	28,2	28,3	27,9
RQ	1,00	0,46	0,97	0,77	0,78	0,46	0,42	0,38	1,08	0,77	0,67	0,82

CHUK					siRNA				RF			
	N	H	N tax	H tax	N	H	N tax	H tax	N	H	N tax	H tax
Ct	28,1	29,1	27,6	29,0	27,4	27,8	27,7	27,9	27,6	28,7	28,9	28,7
RQ	1,00	0,34	0,86	0,44	1,22	0,77	0,57	0,69	0,99	0,44	0,36	0,39

CRADD					siRNA				RF			
	N	H	N tax	H tax	N	H	N tax	H tax	N	H	N tax	H tax
Ct	29,6	29,4	29,5	29,9	29,3	29,0	29,8	29,7	29,4	29,5	30,0	29,7
RQ	1,00	0,80	0,65	0,65	0,92	0,99	0,38	0,57	0,84	0,73	0,49	0,55

NFKBIE					siRNA				RF			
	N	H	N tax	H tax	N	H	N tax	H tax	N	H	N tax	H tax
Ct	29,7	29,7	28,4	28,5	28,9	29,0	28,4	28,5	29,1	29,1	28,4	27,9
RQ	1,00	0,67	1,47	1,79	1,26	1,04	1,05	1,32	1,11	0,99	1,51	1,99

NFKBIZ					siRNA				RF			
	N	H	N tax	H tax	N	H	N tax	H tax	N	H	N tax	H tax
Ct	28,0	29,5	28,0	28,6	27,0	27,7	28,4	28,6	27,4	28,0	28,6	28,5
RQ	1,00	0,25	0,62	0,53	1,48	0,78	0,32	0,38	1,08	0,64	0,39	0,40

PEA15					siRNA				RF			
	N	H	N tax	H tax	N	H	N tax	H tax	N	H	N tax	H tax
Ct	23,4	23,9	22,3	23,1	23,3	23,5	23,1	23,3	23,0	23,5	22,9	22,8
RQ	1,00	0,49	1,29	0,97	0,79	0,59	0,53	0,61	0,93	0,62	0,83	0,85

PMAIP1					siRNA				RF			
	N	H	N tax	H tax	N	H	N tax	H tax	N	H	N tax	H tax
Ct	28,4	29,0	25,8	26,9	27,1	27,2	26,3	26,2	27,8	28,1	26,8	26,6
RQ	1,00	0,46	3,68	2,18	1,81	1,52	1,85	2,59	1,12	0,79	1,88	1,97

PYCARD					siRNA				RF			
	N	H	N tax	H tax	N	H	N tax	H tax	N	H	N tax	H tax
Ct	29,0	29,2	30,5	31,3	29,3	29,7	31,0	30,6	28,5	29,0	30,7	30,5
RQ	1,00	0,63	0,21	0,16	0,59	0,41	0,11	0,19	1,01	0,67	0,20	0,20

RELA					siRNA				RF			
	N	H	N tax	H tax	N	H	N tax	H tax	N	H	N tax	H tax
Ct	25,0	25,0	24,4	24,4	24,6	25,0	24,4	24,9	24,6	24,6	24,5	24,2
RQ	1,00	0,73	0,92	1,23	0,99	0,64	0,66	0,65	0,99	0,88	0,88	1,06

RELB					siRNA				RF			
	N	H	N tax	H tax	N	H	N tax	H tax	N	H	N tax	H tax
Ct	30,3	29,5	27,7	28,0	28,7	28,1	26,8	27,2	29,2	29,1	27,5	27,8
RQ	1,00	1,21	3,73	4,05	2,21	2,91	4,92	4,99	1,55	1,53	4,37	3,39

REL					siRNA				RF			
	N	H	N tax	H tax	N	H	N tax	H tax	N	H	N tax	H tax
Ct	30,9	30,8	31,0	31,5	30,8	30,5	30,6	31,4	30,9	30,5	31,8	31,2
RQ	1,00	0,78	0,61	0,56	0,84	0,91	0,56	0,43	0,76	0,89	0,35	0,48

RIPK1					siRNA				RF			
	N	H	N tax	H tax	N	H	N tax	H tax	N	H	N tax	H tax
Ct	27,8	27,9	26,8	27,7	27,6	27,7	26,9	27,5	27,3	28,0	27,4	27,1
RQ	1,00	0,65	1,19	0,86	0,83	0,67	0,78	0,74	0,98	0,56	0,79	0,93

RIPK2					siRNA				RF			
	N	H	N tax	H tax	N	H	N tax	H tax	N	H	N tax	H tax
Ct	28,1	28,0	26,9	27,8	27,6	27,9	27,2	27,6	27,8	28,5	28,0	27,3
RQ	1,00	0,75	1,47	1,00	1,07	0,77	0,83	0,85	0,90	0,52	0,70	1,07

TA-NFKBH					siRNA				RF			
	N	H	N tax	H tax	N	H	N tax	H tax	N	H	N tax	H tax
Ct	31,9	32,1	30,4	32,1	31,1	31,6	30,5	31,0	31,7	31,4	31,3	31,3
RQ	1,00	0,64	1,75	0,75	1,32	0,82	1,23	1,14	0,88	0,94	0,94	0,92

TBK1					siRNA				RF			
	N	H	N tax	H tax	N	H	N tax	H tax	N	H	N tax	H tax
Ct	28,0	28,6	27,8	28,4	27,6	27,9	28,0	28,3	28,2	28,2	28,4	28,7
RQ	1,00	0,44	0,70	0,59	0,95	0,67	0,43	0,47	0,62	0,58	0,45	0,35

TNFRSF10A					siRNA				RF			
	N	H	N tax	H tax	N	H	N tax	H tax	N	H	N tax	H tax
Ct	27,5	28,0	26,5	27,9	26,8	27,2	26,4	27,0	27,0	27,6	27,1	27,6
RQ	1,00	0,50	1,26	0,61	1,27	0,81	0,96	0,85	1,01	0,62	0,81	0,56

TNFRSF10B					siRNA				RF			
	N	H	N tax	H tax	N	H	N tax	H tax	N	H	N tax	H tax
Ct	25,9	25,9	25,0	25,6	25,4	25,5	24,8	25,3	25,3	25,7	25,2	25,1
RQ	1,00	0,72	1,18	1,01	1,08	0,85	0,96	0,90	1,08	0,75	0,98	1,06

TNFRSF1A					siRNA				RF			
	N	H	N tax	H tax	N	H	N tax	H tax	N	H	N tax	H tax
Ct	24,3	23,6	23,8	24,9	23,7	24,0	23,6	24,2	23,3	24,0	24,4	23,8
RQ	1,00	1,09	0,89	0,54	1,15	0,80	0,69	0,65	1,43	0,83	0,57	0,80

TNFRSF1B					siRNA				RF			
	N	H	N tax	H tax	N	H	N tax	H tax	N	H	N tax	H tax
Ct	30,5	31,3	32,5	33,8	30,0	29,9	32,9	32,6	29,8	30,9	32,7	32,9
RQ	1,00	0,40	0,16	0,08	1,05	0,94	0,08	0,14	1,15	0,51	0,13	0,11

TNFRSF21					siRNA				RF			
	N	H	N tax	H tax	N	H	N tax	H tax	N	H	N tax	H tax
Ct	23,7	25,6	25,5	26,9	23,1	23,1	25,3	25,4	23,5	25,0	25,8	26,5
RQ	1,00	0,20	0,18	0,09	1,15	1,03	0,15	0,18	0,83	0,27	0,15	0,09

TNFSF10					siRNA				RF			
	N	H	N tax	H tax	N	H	N tax	H tax	N	H	N tax	H tax
Ct	31,8	31,0	32,3	31,5	30,9	31,5	32,5	32,6	31,4	30,7	32,7	31,1
RQ	1,00	1,15	0,44	1,00	1,38	0,76	0,26	0,33	0,94	1,36	0,33	0,94

TRADD					siRNA				RF			
	N	H	N tax	H tax	N	H	N tax	H tax	N	H	N tax	H tax
Ct	29,1	28,8	29,1	29,0	29,1	28,9	28,8	29,2	29,2	28,7	28,9	28,7
RQ	1,00	0,86	0,64	0,87	0,73	0,75	0,53	0,55	0,69	0,90	0,70	0,80
