# Supplementary material for: Anti-apoptotic role of HIF-1 and AP-1 in paclitaxel exposed breast cancer cells under hypoxia
Source: Mol Cancer. 2010 Jul 13;9:191. doi: 10.1186/1476-4598-9-191 (PMC3098009; doi:10.1186/1476-4598-9-191)
Supplement: Additional file 3 — Effect of c-jun silencing on c-jun expression. MDA-MB-231 cells were transfected 24 h with c-jun siRNA (siRNA) or RISC-free control siRNA (RF) (50 nM). Cells were then incubated with or without paclitaxel (tax, 50 μM) under normoxic (N) conditions for 16 hours. After transfection and incubation, total RNA has been extracted and retro-transcribed in cDNA. A real time PCR has been performed with specific primers for C-JUN and for RPL13A, a house-keeping gene. Results are expressed in induction level by comparison with the reference condition, normoxia. [file 1476-4598-9-191-S3.PDF]

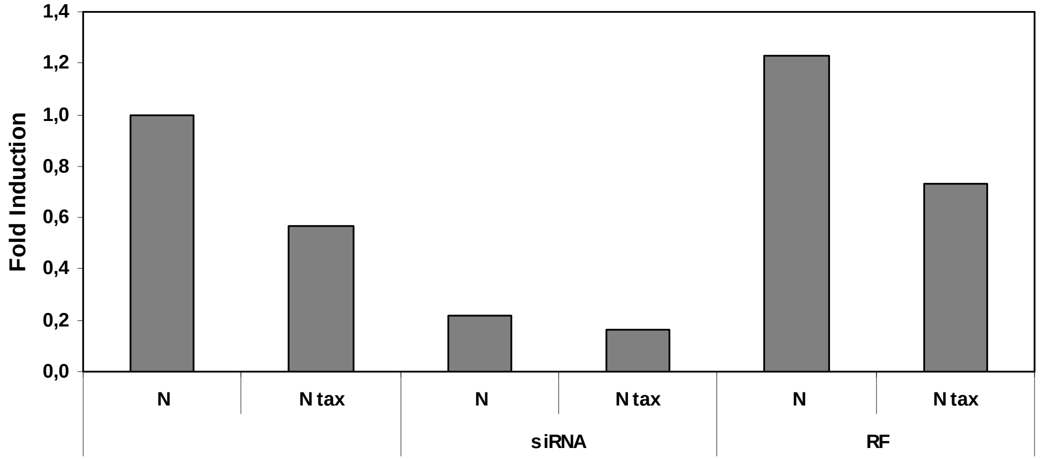


**Additional file 3**

Effect of c-jun silencing on c-jun expression. MDA-MB-231 cells were transfected 24 h with c-jun siRNA (siRNA) or RISC-free control siRNA (RF) (50nM). Cells were then incubated with or without paclitaxel (tax, 50µM) under normoxic (N) conditions for 16 hours. After transfection and incubation, total RNA has been extracted and retro-transcripted in cDNA. A real time PCR has been performed with specific primers for C-JUN and for RPL13A, a house-keeping gene. Results are expressed in induction level by comparison with the reference condition, normoxia.
